# Tibial tubercle osteotomy for access during revision knee arthroplasty: Ethibond suture repair technique

**DOI:** 10.1186/1471-2474-9-98

**Published:** 2008-06-30

**Authors:** Crawford R Deane, Nicholas A Ferran, Adel Ghandour, Rhidian L Morgan-Jones

**Affiliations:** 1 Department of Trauma and Orthopaedics, University Hospital of Wales, Heath Park, Cardiff, CF14 4XW, UK

## Abstract

**Background:**

Tibial Tubercle Osteotomy has shown much promise in revision total knee replacement. Methods of repair previously described include screw and wire fixation. Both methods have significant complications.

**Methods:**

This article describes suture fixation of the osteotomy using Ethibond sutures placed medially with a lateral periosteal hinge.

**Results:**

This method of fixation relies upon an adequate osteotomy segment including the entire insertion of the patella tendon. The lateral periosteal hinge is maintained and adds to the stability of the construct. A minimum of two number 5 Ethibond sutures are passed medially through drill holes to secure the osteotomy segment. No post-operative immobilisation is required.

**Conclusion:**

Ethibond sutures provide adequate fixation of the tibial tubercle osteotomy segment in revision knee arthroplasty with reduced risk of complication as compared to conventional fixation methods.

## Background

Exposure in revision total knee arthroplasty is challenging. Tibial tubercle osteotomy is a technique gaining popularity in knee revision surgery which allows exposure and access to the medullary canal of the tibia, with reduced risk of extensor lag compared to techniques involving the quadriceps [1]. The tibial tubercle is transected in the coronal plane maintaining the lateral periosteal sheath in order to preserve blood supply to the osteotomy and also to provide mechanical support in fixation.

Two methods of fixation of the osteotomy segment have been described: screw fixation and cerclage wire fixation. Screw fixation is associated with anterior knee pain, necessitating screw removal, osteotomy segment fracture, and tibial shaft fracture [2]. Cerclage wire fixation usually requires passage of three wires through the tibia from medial to lateral, and encircling the osteotomy segment. A step cut, on the proximal edge of the osteotomy, is often used to reduce the incidence of proximal migration. Wire fixation is associated with soft tissue injury caused by the tails of the wires after they are tied, proximal migration of the osteotomy segment, and anterior knee pain [3].

Suture fixation of the TTO has been developed by the senior author to reduce the complications associated with traditional fixation techniques in the setting of the difficult revision knee arthroplasty. This paper describes the method of suture repair of TTO as developed and used at our institution.

## Results

All patients undergo a process of informed consent prior to surgery. Under general or spinal anaesthetic, the patient is placed in the prone position, and a mid-thigh tourniquet is applied and inflated to 300–350 mmHg. A lateral support and bolster are used to maintain the knee in approximately 90° flexion. A standard aseptic preparation and drape is used.

Through a medial parapatellar approach the extensor retinaculum is incised from the apex of the quadriceps tendon to the tibial tubercle. Where significant further release is required the tibial tubercle is exposed entirely.

**Figure 1:**
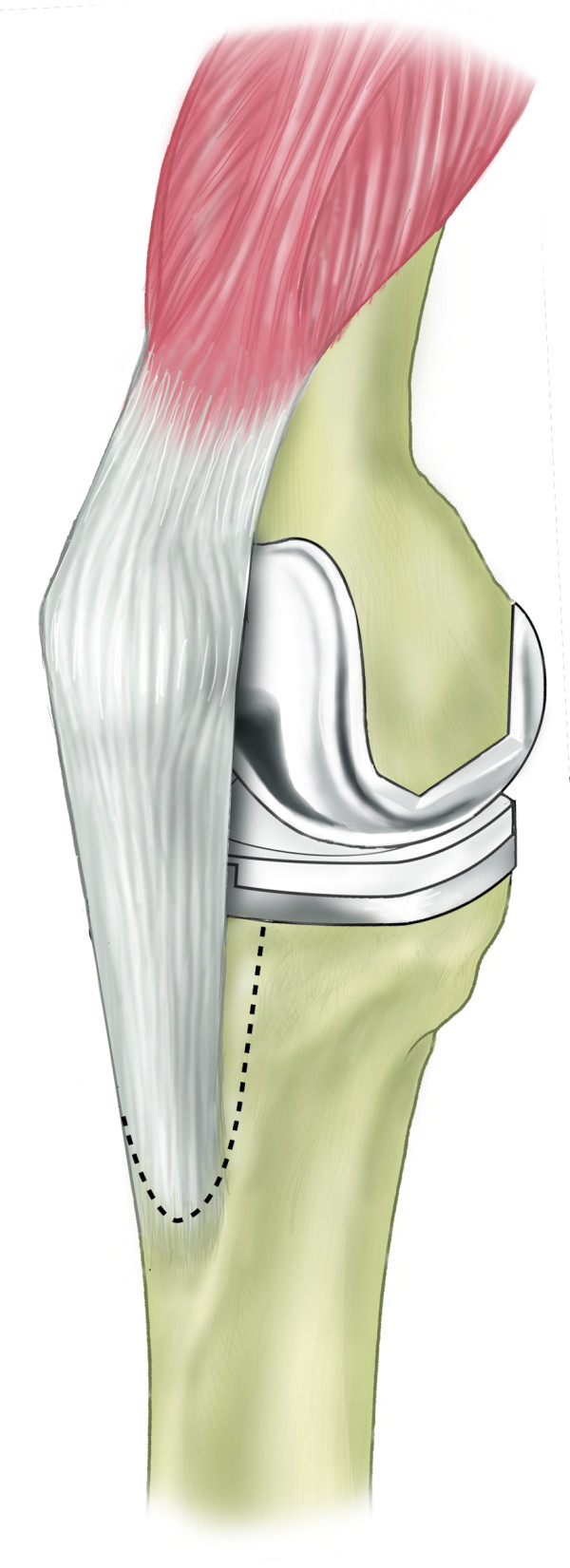
Schematic diagram demonstrating extent of osteotomy.

**Figure 2:**
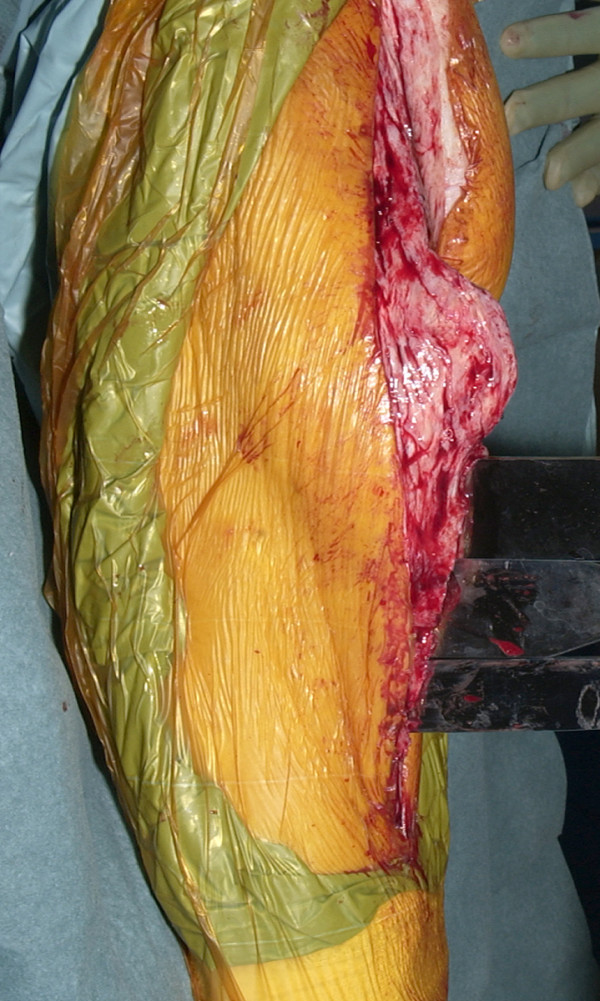
Osteotomy created with use of osteotomes.

**Figure 3:**
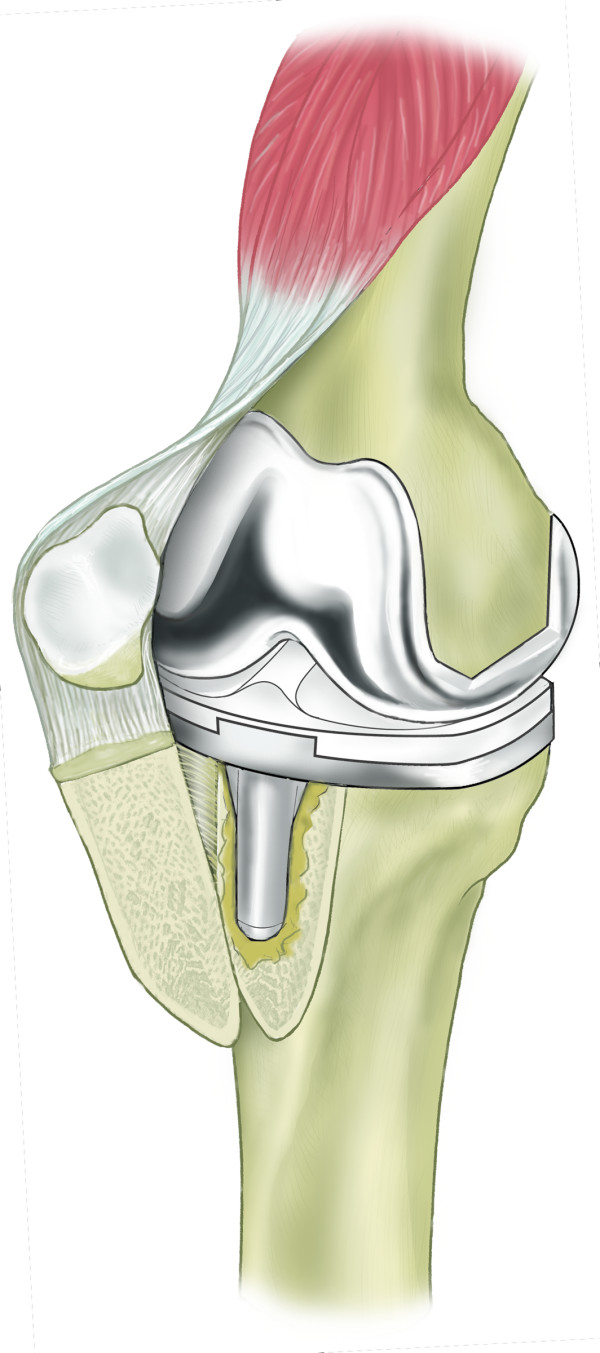
Schematic diagram demonstrating lateral periosteal hinge allowing reflection of osteotomy segment.

**Figure 4:**
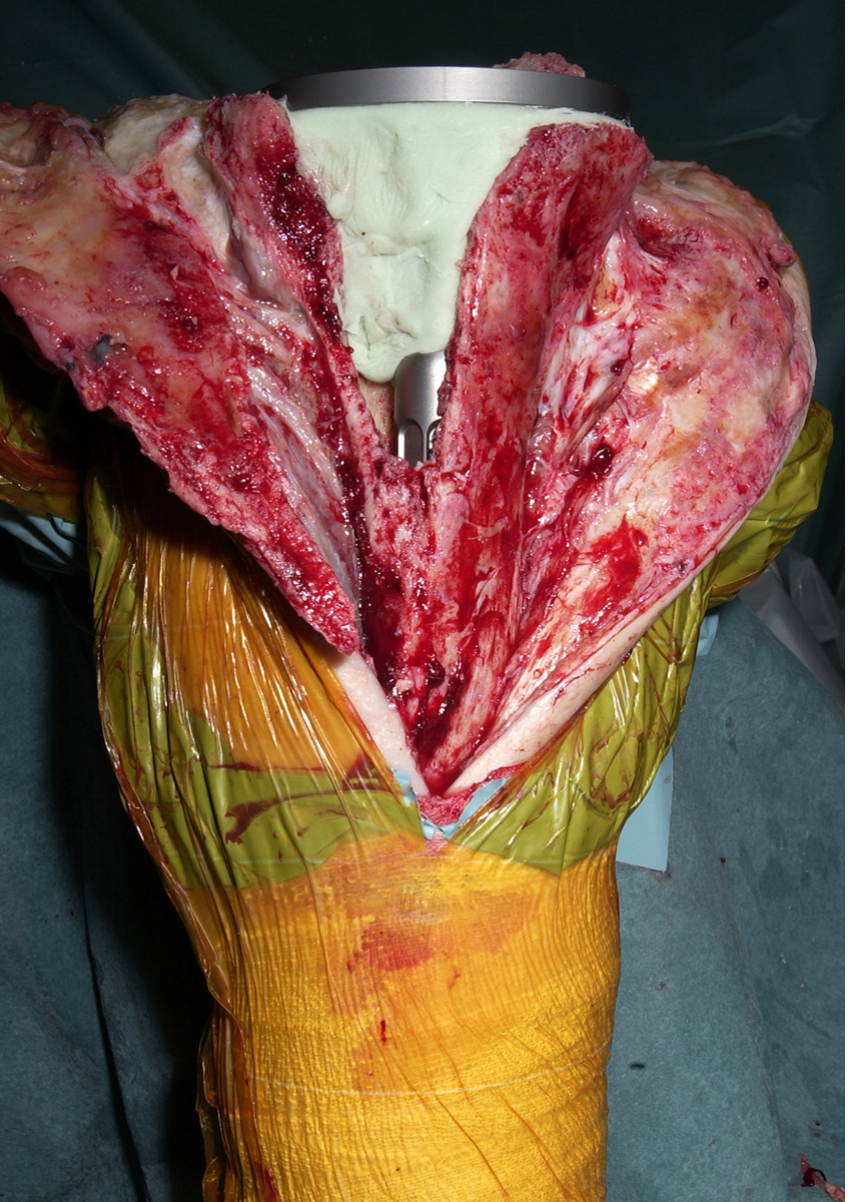
Exposure gained by use of osteotomy.

The tibial tubercle osteotomy segment is created using an osteotome medially to provide a maximum thickness of approximately 1 cm. Care is taken to include all of the insertion of the patellar tendon (Figure 1). The osteotome is passed through the medial cortical bone, the cancellous bone and partially through the lateral cortical bone (Figure 2). The lateral periosteum is left intact to act as a hinge for the osteotomy segment. No proximal or distal steps are made. The osteotome is then used to lever the segment from the tibia (Figure 3), providing access to the medullary canal of the tibia (Figure 4).

Realignment is made under direct vision with the segment usually restored to its initial position. Where it is moved to compensate for patellar malignment the periosteal sheath is partially released and stretched to facilitate realignment prior to making drill holes.

**Figure 5:**
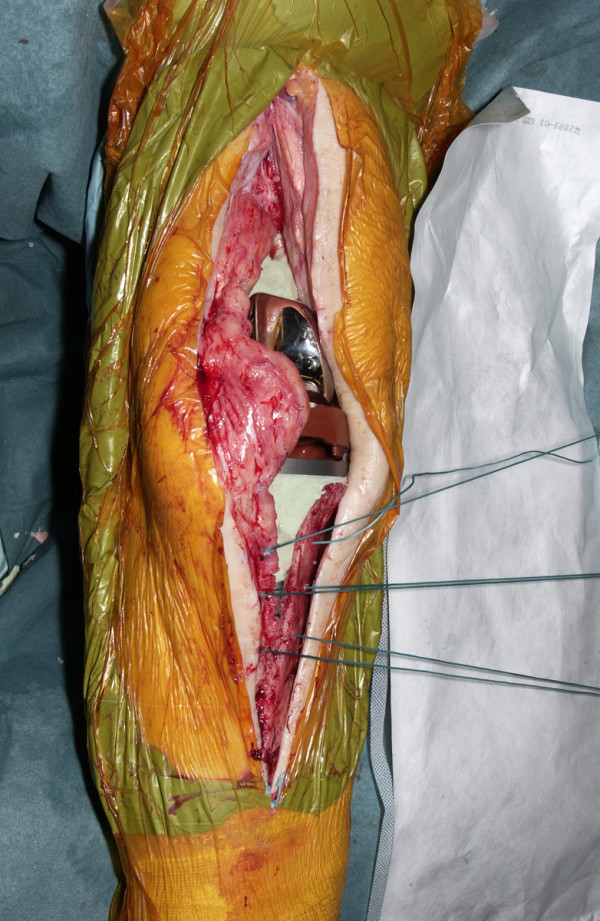
Placement of drill holes and sutures.

Drill holes of 2 mm in diameter are made in the cortex of the osteotomy segment and native medial tibial surface. Drill holes should be positioned carefully to avoid fracture (Figure 5).

The drill holes are positioned so as to allow a 55 mm, 1/2 circle tapercut needle to pass through the cortical surfaces of the osteotomy segment and the tibia. Ideally the distance between the two drill holes should be approximately the same as the diameter of the needle. The paired drill holes in the tibia and osteotomy segment are located approximately equidistant from the cut line.

Drill holes are placed at a minimum distance of 1 cm apart vertically on the tibial surface. The number of sutures used therefore depends on the size of the osteotomy segment and its profile. A minimum of two sutures are required for adequate fixation however three sutures are ideal.

**Figure 6:**
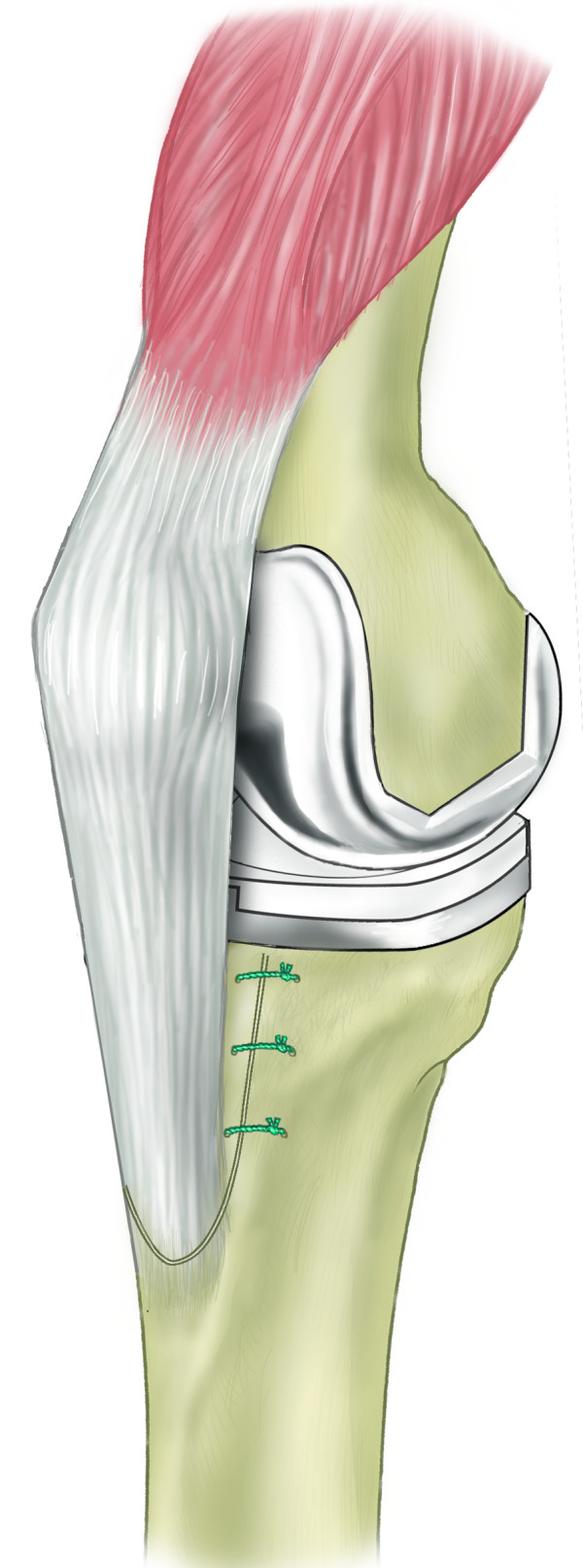
Schematic diagram of repaired osteotomy.

Number 5 Ethibond (Ethicon, Somerville, NJ) braided polyester sutures are used to secure the osteotomy. A single pass through each drill hole is used with the knot placed medially to reduce prominence and discomfort when kneeling (Figure 6).

Ethibond is preferred for its high tensile strength and ease of handling. Ethibond is less cumbersome than wires and less prominent than screws. The 55 mm, 1/2 circle, Tapercut needle allows improved bone penetration when passing sutures.

### Post operative care

**Figure 7:**
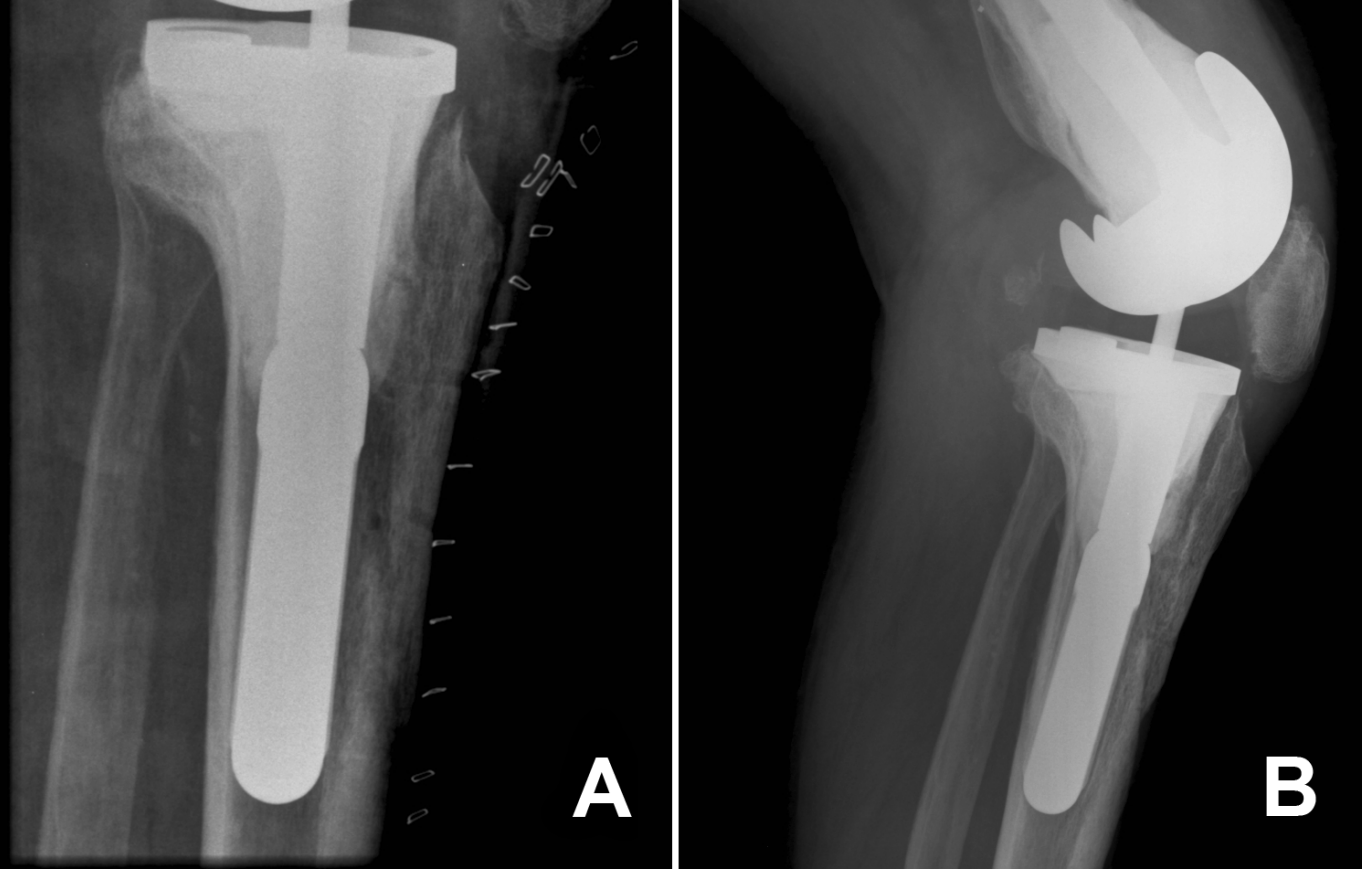
Radiograph of suture repair of tibial tubercle osteotomy immediately post op (A) and at follow-up demonstrating trabeculae crossing the osteotomy site (B).

Patients are encouraged to weight bear with crutches 24 hours post operation, and are assessed by a physiotherapist to ensure safe mobilisation prior to discharge. Patients are also prescribed a course of outpatient physiotherapy focussing on range of motion and strengthening of the musculature around the knee. Follow-up regimen does not differ from other revision arthroplasty patients (Figure 7).

## Discussion

Tibial tubercle osteotomy has a lower incidence of extensor lag compared to exposures utilising the quadriceps such as quadriceps turndown [1]. While tibial tubercle osteotomy provides excellent access in revision knee arthroplasty, traditional methods of fixation of the osteotomy segment are not without complication.

van den Broek et al. reported their results of 37 revision knee arthroplasty patients managed with a step cut TTO fixed with screws; 5 patients had reoperation – 1 for refixation of migrated fragment, 1 for reconstruction of the patellar tendon following an avulsion and 3 for removal of painful screws [4].

Hocking et al. reported on 49 cases of TTO with cerclage wire fixation. One patient required revision of a migrated osteotomy segment and one had an intra-operative fracture of the osteotomy segment [5]. Mendes et al. reported on 67 revision knee arthroplastys treated with TTO fixed with cerclage wires. There were 2 undisplaced tibial stress fractures and 1 fibrous union. Two patients required removal of wires due to pain [6].

Suture fixation of the osteotomy has several advantages over other fixation methods. Placement of sutures is less difficult than navigating screws to avoid the tibial revision component. Ethibond offers easier handling than wire and has excellent tensile strength. With an intact lateral periosteal sleeve it provides adequate fixation in the setting of revision knee arthroplasty. In our experience we have never had to revise osteotomy segment fixation with sutures.

Ethibond sutures offer less anterior prominence than both screws and wires in order to reduce the incidence of anterior knee pain and we have not had to re-operate to remove troublesome or prominent sutures. Stresses on the osteotomy segment are also more widely distributed with sutures than with screws reducing the likelihood of fracture. We believe medial suture fixation, enhanced by an intact lateral periosteal hinge, provides a mechanically robust fixation and thus a step cut is deemed unnecessary, eliminating the stress riser effect associated with this method. We have never encountered a fracture related to this procedure.

Suture repair of tibial tubercle osteotomy does however rely on the integrity of the lateral periosteal sleeve for stability and is therefore at risk of migration of the osteotomy segment. We have not experienced a case in which the integrity of the periosteum and lateral soft tissues was inadequate such that it compromised fixation. Migration is also a recognised complication of the other methods of fixation and we have not had to revise fixation due to migration.

Ethibond is known to be biocompatible with minimal tissue reactivity but the possibility of an inflammatory response remains although this has not been our experience [7].

## Conclusion

Ethibond sutures provide an alternative method of repair of tibial tubercle osteotomy when used for access in revision knee arthroplasty. This technique is easier to perform with reduced risk of re-operation and fracture as compared with screw and cerclage wire fixation.

## Methods

The tibial tubercle osteotomy segment is created using an osteotome medially to provide a maximum thickness of approximately 1 cm. Care is taken to include all of the insertion of the patellar tendon (Figure 1). The osteotome is passed through the medial cortical bone, the cancellous bone and partially through the lateral cortical bone (Figure 2). The lateral periosteum is left intact to act as a hinge for the osteotomy segment. No proximal or distal steps are made. The osteotome is then used to lever the segment from the tibia (Figure 3), providing access to the medullary canal of the tibia (Figure 4).

The osteotomy segment is realigned under direct vision. Restoration to initial position is used in most cases; however, in cases of gross disorder of patellar alignment, such as patella baja, the osteotomy is purposely relocated to correct malalignment. Relocation of the patella requires the lateral periosteal hinge to be partially released and relies on the elasticity of the tissues for mobilisation.

Drill holes of 2 mm in diameter are made in the cortex of the osteotomy segment and native medial tibial surface. Drill holes should be positioned carefully to avoid fracture (Figure 5).

The drill holes are positioned so as to allow a 55 mm, 1/2 circle tapercut needle to pass through the cortical surfaces of the osteotomy segment and the tibia. Ideally the distance between the two drill holes should be approximately the same as the diameter of the needle. The paired drill holes in the tibia and osteotomy segment are located approximately equidistant from the cut line.

Drill holes are placed at a minimum distance of 1 cm apart vertically on the tibial surface. The number of sutures used therefore depends on the size of the osteotomy segment and its profile. A minimum of two sutures are required for adequate fixation however three sutures are ideal.

Number 5 Ethibond (Ethicon, Somerville, NJ) braided polyester sutures are used to secure the osteotomy. A single pass through each drill hole is used with the knot placed medially to reduce prominence and discomfort when kneeling (Figure 6).

## Competing interests

The authors declare that they have no competing interests.

## Authors' contributions

RMJ conceptualised the technique. CD performed the literature review. CD, NF and AG were responsible for manuscript preparation and editing.

## Consent

Patient consent is on file for use of operative images for publication and presentation.

## Pre-publication history

The pre-publication history for this paper can be accessed here:


